# Kamri; or, Paraplegia

**Published:** 1887-10

**Authors:** R. W. Burke

**Affiliations:** Station Chief Vet. Hospital, Jubbulpore, India


### ART. XXXIII.—KAMRI, OR PARAPLEGIA.

BY R. W. BURKE, A. V. D.
*Station Chief Vet. Hospital, Jubbulpore, India.*

The word "*kamri*" is derived from the Persian word *kamer*, which signifies the loins; kamri, therefore, denoting disease of the loins. A somewhat similar disease in man is called "ardhang," or more literally "adhangi," *adha* meaning half, *áng* body, *i.e.*, having the use of half the body, or affecting half the body only.

*Etiology.*—Kamri may be brought on (1) from exposure to damp and inclement weather; (2) by dietetic causes; (3) by surgical conditions; (4) and it may be a result of many specific fevers, especially one form of anthrax, rabies, surra, kákke, and other diseases.

(1) Exposure is one of the most frequent causes of kamri in India. It has been generally noted that picketing horses outside their stables for a single night, will often suffice to bring on an attack of kamri. This is especially remarked during damp seasons, and is explained on the "chill theory," according to which the nervous system, in endeavoring to adapt itself to the needs of the body, exposed to the great and violent changes of temperature said to occur during certain seasons in India, breaks down and becomes disorganized; that is to say, kamri is essentially a disease of the nervous system caused by exposure to climatic changes. Chill is probably a wrong term to use, for the breakdown is nearly as often occasioned by exposure to excessive heat as to excessive cold; and considering that we know practically nothing of what occurs in the body when exposed to a temperature above its own, the theory cannot be summarily rejected. From a large number of observations made by the writer in these cases, it has seemed, first, that the changes of temperature in certain seasons are sudden and violent; and, secondly, that the connection between these variations of temperature and perhaps the malarious character of localities in which they are often observed is such as to lead to the conclusion that if not in themselves the cause of the disease, they undoubtedly have a most intimate connection with it and suggest precautionary measures in localities where kamri is most frequent. Much may, indeed, be done in all cases by avoiding exposure to the above-mentioned extreme variations of temperature, and by the maintenance of "tone" in the nervous system. As a rule, most cases occur towards the close of the rainy season, and during the winter, only a few cases appearing in the hot months. Enzootic visitations are quite exceptional, and in none of these is fever-height indicative of a specific origin—in fact, one peculiarity of this disease is the absence of any febrile symptoms, the patients maintaining their appetite throughout and showing no signs of illness, save the peculiarity of gait noticed in these cases.

(2) There are also certain dietetic causes in operation which produce kamri both in horses and in men, and which have been proved sufficient to induce this disease when experimentally given to animals, or taken by human beings, as food. A variety of Indian pulse, which is found to be identical with the *Lathyrus sativus*, and known as the kussari, is fraudulently mixed with grain sold in the bazaars in India, and is a frequent cause of paralysis in horses when eaten in sufficiently large quantity to have effect. It is grown as a cold-weather crop on land which will raise no other kind of pulse—chiefly clayey soils and land submerged in the rainy season, which hardens during the cold weather almost to the consistency of stone and splits up into long, deep fissures. It occasionally grows in rice-fields whilst the rice stubble is still standing. Chemically it is exceptionally rich in nitrogenous constituents, and this may account for its tendency to produce paralysis.

(3) Surgical Conditions.—(*a*) *Reflex Kamri.*—Clinical observation teaches us that not merely motory impressions, but those also which cause sensations, may be reflected; so that the impression of one part is experienced by sensation in another. Impressions on the ultimate distribution of one nerve produce sensations in parts supplied by another nerve, or by another branch of the same nerve. Thus in shoulder lameness, due to disease of the liver and in lameness behind, due to renal and cystic disease, calculi, etc., the sensations cannot be referred to direct nervous communication, but to an influence reflected, probably from the spinal centre only. In intestinal concretions, and in acute colic in the lower animals, we are familiar with lameness from pain reflected to the extremities. Experimental facts show that there are reflex inhibitory centres in the cord. We are acquainted with nerves whose action consists in the inhibition of the action of other nerves; and so pathologists have been induced to look upon reflex paralysis and reflex inhibition as very similar processes. (*b*) *Traumatic Kamri.*—Kamri is sometimes produced in its worst form from injury to the spine.

(4) Symptoms resembling those of ordinary kamri may be seen accompanying the course of certain specific diseases, but especially that of anthrax, rabies, surra, and other diseases. We may consider these *seriatim:*—(*a*) We are familiar with a form of paralysis of the hinder quarters, which occurs in cases of rabies in the horse, but especially toward the latter stages of that malady; and this form of the disease simulates kamri in the horse when the history of the case is not known. Diagnosis is generally easy when we have the previous history of the case, and when other symptoms, absent in kamri, have been remarked in the case preceding the paralysis. (*b*) One form of anthrax, attended with paralysis of the hind quarters, may be mistaken for kamri, but is distinguishable from it by (1) the course of the disease; (2) the concomitant symptoms of anthrax, which are never witnessed in kamri; and (3) the presence of the *bacillus anthracis* in the blood and other tissues of the patient.

(*c*) Dr. Wallace Taylor made the observation, in 1880, that outbreaks of "beri-beri" in man were frequently associated with outbreaks of paralysis in animals—but especially in ponies and mules in Burmah, China and adjoining countries—and that this disease of animals had a pathological relation to that seen in human beings, in that the blood of affected animals showed, on examination, the same organisms—*bacilli*—as Dr. Taylor had described in "beri-beri" of man. The subject of beri-beri has attracted considerable attention in India and elsewhere, and every day new and hitherto imperfectly understood diseases are coming under its nomenclature. Sir J. Fayner even suggests that pernicious anæmia is probably the same disease in Europe. Cases of œdema of obscure origin, as well as other diseases so often met with in practice in India, might also be shown to be but different forms of beri-beri. One fact is noteworthy in Dr. Taylor's account, namely, change of situation and climate always brings about an improvement in these cases; and this fact has been noted also in animals, as Dr. Taylor mentions both an acute and a chronic form of paralysis, and it is the latter cases which benefit mostly by a change of situation. (*d*) Veterinary Surgeon Steel noted in the Burmah outbreak, which he was deputed to investigate in 1884, that paralysis of the hinder quarters was remarked in many cases of that disease in mules; and he has since seen the same disease in animals at Poonah, where this symptom of paralysis was seldom absent.

*Diagnosis.*—I think the etiology of the disease shows that the subject of kamri in the horse—whether true or symptomatic kamri—is at once wide and interesting, and therefore worthy of our closest consideration. We must not, however, confound the simple forms of kamri with those due to specific causes, which are readily distinguishable by the course and concomitant symptoms, the presence of the specific organisms in the blood and other tissues; the influence of treatment, etc. The paraplegia accompanying the course of specific fevers—anthrax, rabies, surra, etc—is not a *neurosis*, but is without doubt symptomatic, arising from reflex irritation of some remote disturbance, in this instance probably some blood-vascular derangement of congestive character. It is obvious that the spinal cord being a complex organ, any reflex irritation arising from it would be more or less generally manifested, thus explaining the posterior paralysis sometimes noted in the course of these diseases. It is generally transient in character, of varying degrees of severity, and usually passes off on the disappearance of the other symptoms common to the disease. The paralysis in kamri comes on quite independently of any febrile disturbance, and always runs a prolonged course, the symptoms lasting for many months or even years. In fact, general belief stamps the disease to be, as a rule, incurable through life; hence all animals affected with kamri, in the old stud days, were sold by public auction, as unfit for the service. The specific fevers in which paralytic spmptoms are noted, are contagious and enzootic, while kamri proper is not.

*Symptoms.*—A horse affected with kamri stands in the stall with his hind legs under him, *i.e.*, places them somewhat forward under the belly and parallel to each other, instead of one leg before the other as in health. He changes his position now and then, and again stands as above mentioned. In backing he does so with difficulty, and drags his hind legs under him, and, if any force is used, even sometimes goes down on his haunches. In trotting he has a rolling action behind, and the hind legs swing from side to side, especially in going up or down hill, which is noticeable from the hips downwards. In turning at a trot on the left, the animal swings the off hind leg outwards, and the near hind leg when turning on the right side. Some severe cases have a difficulty in rising in the stall, and, except in cases of kamri from injury, they evince no pain on pressure to the loins. These are the symptoms met with in ordinary kamri, and in ninety-nine per cent. of the cases seen; the more acute symptoms of paralysis being referable chiefly to those of a specific origin, or to neglect of treatment in the early stages of the simple cases.

*Prognosis.*—I think that it may be stated generally that a person should not purchase a horse for at least twelve months after amelioration of the acute symptoms, and that he ought not to do so whilst any weakness in the animal's action is noticed, whatever time has elapsed since apparent recovery. The prognosis is more hopeful in young than in very old animals.

*Treatment.*—This should be both curative and preventive in kind. The treatment which is found most efficacious in practice is the internal administration of nux vomica, commencing with 20 gr. doses, and gradually increasing the amount to two drachms each dose, given twice daily till improvement is noticed. In some cases the iodide of potassium will be found beneficial, with iron and other tonics. It is always well to administer a physic previous to commencing the treatment by more specific agents. When symptoms simulating those of kamri proper follow in the course of any specific fever, the same treatment will be indicated as is found beneficial in the management of that particular fever ; we must, in fact, treat the disease, and not the symptom, in this instance.

*Pathological Anatomy.*—There are two kinds of changes of the cord principally associated with cases of kamri ; the atrophic and the hydræmic or œdematous, which may be found in varying degrees of severity in different cases, depending on the stage of the disease at which the *post-mortem* examination is made. The above changes are, doubtless, of secondary origin, the result of the disturbance of function, and afford no indication of the morbid influence by which the function is disturbed. The one almost solitary fact which we have to guide us in seeking for the seat of the disease is the change in the irritability of the motor nerves of the hinder quarters. This proves beyond a doubt that the nutrition of these nerves is changed. But such a change in the nerves suggests a similar in the motor nerve-cells of the spinal cord. Of these the motor axis-cylinders are the processes, and share the changes in nutrition of the parent cell. Moreover, the loss of power, or paralysis itself, indicates that there is a morbid state of the gray matter, since we know that only nerve-cells can liberate the energy which causes motor power. We may feel sure, therefore, that the motor nerve-cells of the cord and the fibres proceeding from them are in a morbid state. But here we must stop. Whether the morbid condition arises primarily in the cord, or descends from higher centres, we cannot tell; and opinions which may be given, that this or that part of the brain is the seat of the disease, would belong, in the present state of our knowledge, to the region of unsupported theory. We are not justified in going for the seat of the disease beyond the spinal axis, including the grey matter of the pons and medulla, in which a morbid state is undoubtedly indicated. Of the nature of the change in the nerves and their centres we know almost nothing. The enduring alteration in function proves some change in their nutrition; but the fact that this change remains limited to weakened power, and frequently passes away entirely or becomes greatly improved under appropriate treatment, proves that it is comparatively slight in degree, and probably limited to such fine molecular changes as could not be recognized by any means of investigation at present at our disposal. The functional alteration in the motor nerves is at present the only pathological indication we possess. But functional disturbance in the nervous system often indicates vasomotor disturbance. The vasomotor symptoms, therefore, that are now and then witnessed in some cases, are clearly secondary in time; and we are not justified in assuming that they are in any way a special feature of the disease. So also the leucocytal aggregation about the vessels, engorgement of them, etc., which have been noted in a few severe cases, are slight and variable traces of similar disturbances in the spinal cord.

